# A Comprehensive Review on the Biosynthesis of Tropane Alkaloids

**DOI:** 10.3390/molecules31172951

**Published:** 2026-08-23

**Authors:** Shiyu Wan, Yafei Zhang, Shengyu Yang, Zhihua Liao, Fei Qiu

**Affiliations:** Integrative Science Center of Germplasm Creation in Western China (Chongqing) Science City, School of Life Sciences, Southwest University, Chongqing 400715, China; wsy1201@email.swu.edu.cn (S.W.); iron123@email.swu.edu.cn (Y.Z.); yangshengyu@email.swu.edu.cn (S.Y.); zhliao@swu.edu.cn (Z.L.)

**Keywords:** tropane alkaloids, biosynthetic pathway, convergent evolution, gene loss, synthetic biology, metabolic engineering

## Abstract

Tropane alkaloids (TA) constitute a class of plant specialized metabolites with important pharmaceutical applications, including the anticholinergic agents hyoscyamine and scopolamine and the local anesthetic cocaine. Over the past decade, advances in genomics, structural biology, and synthetic biology have substantially revised our understanding of TA biosynthesis, leading to the identification of numerous key biosynthetic enzymes and evolutionary mechanisms. This review comprehensively summarizes current knowledge of TA biosynthesis from precursor formation to structurally diverse end products. We describe the pathway from putrescine to tropinone, the stereoselective metabolic branching mediated by Tropinone Reductases, and the downstream biosynthesis of medicinal tropane alkaloids, calystegines, and cocaine. Particular emphasis is placed on recent discoveries concerning catalytic mechanisms, structural determinants of substrate specificity, metabolic compartmentalization, and the convergent evolution of TA biosynthesis in Solanaceae and Erythroxylaceae. We further integrate advances in genomics, evolutionary biology, and metabolic engineering to highlight emerging strategies for microbial production and pathway redesign. By providing a comprehensive synthesis of recent progress and critical perspectives on unresolved questions, this review offers an updated framework for understanding TA biosynthesis and supports future research in plant specialized metabolism, synthetic biology, and natural product engineering.

## 1. Introduction

**Figure 1:**
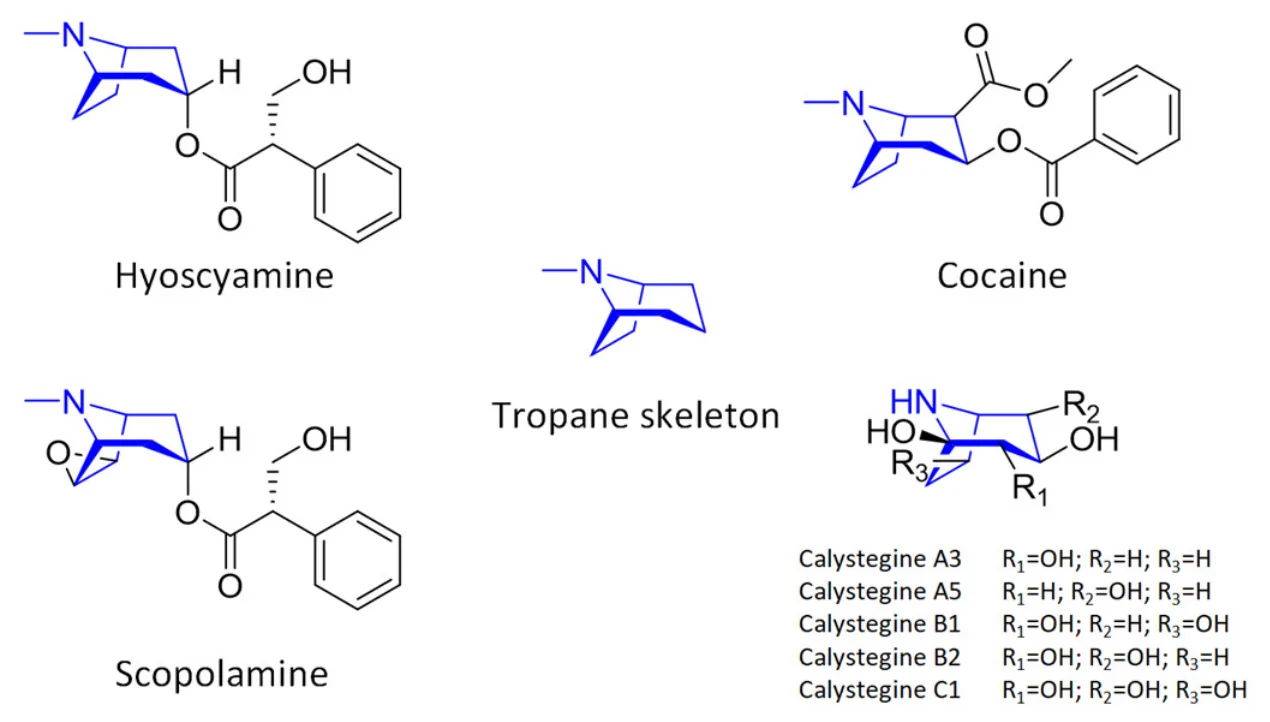
The tropane skeleton and representative structures of tropane alkaloids. The blue moieties highlight the conserved tropane bicyclic scaffold common to tropane alkaloids.

Tropane alkaloids (TA) are a class of structurally distinctive, pharmacologically potent plant specialized metabolites whose distribution across angiosperms exhibits a highly patchy and discontinuous phylogenetic pattern [1,2,3]. These alkaloids share a characteristic tropane bicyclic scaffold, formed by the fusion of a pyrrolidine ring and a piperidine ring, and have thus far been identified primarily in a limited number of plant lineages, including the Solanaceae, Erythroxylaceae, and Convolvulaceae [4,5,6]. Among them, Solanaceae species predominantly accumulate medicinal tropane alkaloids derived from tropine (3α-hydroxytropane), including hyoscyamine (whose racemate is atropine) and scopolamine. As classical anticholinergic agents, these compounds are widely used in clinical practice for preoperative medication, antitussive and bronchodilatory therapy, prevention and treatment of motion sickness, slowing the progression of juvenile myopia, and management of organophosphate poisoning [7,8,9]. In addition, some species produce polyhydroxylated tropane alkaloids derived from pseudotropine (3β-hydroxytropane), namely calystegines, which exhibit potent glycosidase inhibitory activity and are regarded as promising lead compounds for the treatment of metabolic disorders [10,11,12,13]. In contrast, members of the Erythroxylaceae specifically synthesize the 3β-configured tropane alkaloid cocaine, which possesses both excellent local anesthetic properties and potent central nervous system stimulatory activity, making it valuable in clinical local anesthesia and neuroscience research [14,15]. The representative structures of tropane alkaloids are depicted in Figure 1.

Owing to their remarkable pharmacological activities, TA have long served as an important source of natural products for drug discovery and clinical application [4,16]. However, the natural abundance of these compounds—including the anticholinergic agents hyoscyamine and scopolamine in Solanaceae as well as cocaine in Erythroxylaceae—is generally low. Industrial production therefore still relies predominantly on cultivation of medicinal plants followed by extraction from natural sources [17,18,19]. This production strategy is constrained by plant growth cycles, geographical and environmental factors, and agricultural conditions, while the extraction process is labor-intensive and costly, making it increasingly difficult to satisfy the growing pharmaceutical demand [20,21,22]. Although substantial progress has recently been achieved in total chemical synthesis, industrial-scale application remains impractical because of lengthy synthetic routes and high production costs [23,24,25]. Consequently, the development of efficient biosynthetic production platforms for TA through metabolic engineering and synthetic biology has emerged as a major research direction in natural product biosynthesis [26,27,28]. Achieving this goal requires comprehensive elucidation of TA biosynthetic pathways and comprehensive identification of the key enzymes and functional genes responsible for each catalytic step. For decades, the elucidation of TA biosynthesis has therefore been regarded as one of the most representative and challenging problems in the field of plant natural product biosynthesis [29,30].

Beyond their pharmaceutical significance, the unusual phylogenetic distribution of TA has also posed a longstanding question in plant evolutionary biology. In striking contrast to medicinal Solanaceae species that accumulate high levels of TA, major crop species within the same family—including tomato (*Solanum lycopersicum*), potato (*Solanum tuberosum*), and pepper (*Capsicum annuum*)—have completely lost the capacity to synthesize medicinally valuable tropane alkaloids during evolution and domestication [31,32]. Therefore, elucidation of the TA biosynthetic pathway is not only fundamental for metabolic engineering but also provides an excellent model for investigating the origin, evolution, and loss of plant specialized metabolic pathways.

Over the past four decades, rapid advances in molecular biology, genomics, and structural biology have led to a series of major breakthroughs in TA research [27,33]. The discovery of hyoscyamine 6β-hydroxylase (H6H) in the 1980s marked the beginning of the molecular era of TA research [34,35,36]. Following nearly forty years of intensive investigation, the biosynthetic pathway of medicinal tropane alkaloids in the Solanaceae has now been almost completely elucidated [37,38]. More recently, the publication of chromosome-level genome assemblies for multiple TA-producing species has provided unprecedented insights into longstanding evolutionary questions. These studies demonstrated that the patchy distribution of TAs within the Solanaceae primarily resulted from multiple independent losses of core biosynthetic gene clusters after their establishment. Indeed, remnants of several downstream biosynthetic genes are still detectable within syntenic genomic regions of non-medicinal Solanaceae species such as tomato and potato [32,39]. Meanwhile, although the Solanaceae and Erythroxylaceae diverged approximately 129 million years ago, both lineages independently evolved the capacity to synthesize the tropane scaffold, representing a classic example of convergent evolution in plant specialized metabolism [40]. From the initial polyamine methylation and polyketide chain elongation steps to scaffold oxidation, cyclization, and terminal acylation, the two pathways independently recruited distinct ancestral gene families and exhibit substantial differences in catalytic mechanisms, subcellular localization, metabolic compartmentalization, and substrate stereoselectivity [39,41]. Collectively, these findings have greatly expanded our understanding of the biosynthetic mechanisms underlying plant natural products while providing a theoretical framework for the rational design and precision biosynthesis of tropane alkaloids.

**Figure 2:**
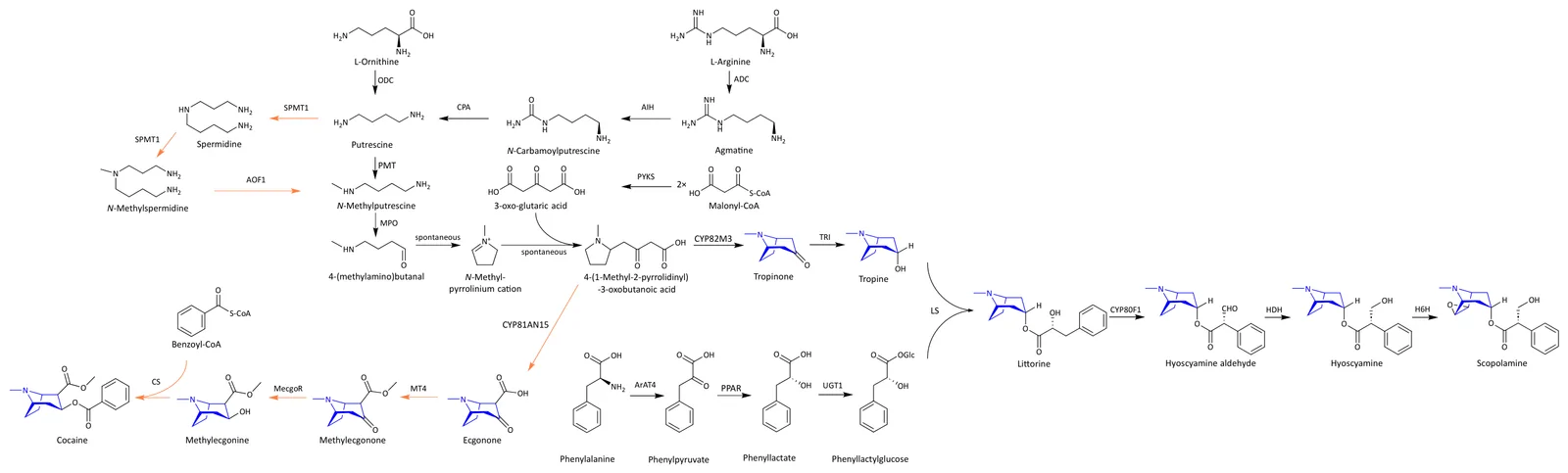
Overview and divergence of tropane alkaloid biosynthetic pathways. The black arrows represent the biosynthetic pathway leading to hyoscyamine and scopolamine in Solanaceae, whereas orange arrows highlight the pathway specific to cocaine biosynthesis in Erythroxylaceae. The scheme summarizes the major enzymatic steps involved in the formation and diversification of the tropane scaffold and highlights the distinct biosynthetic strategies employed by different plant lineages. The blue moieties highlight the conserved tropane bicyclic scaffold common to tropane alkaloids.

In light of the significant advances achieved in recent years regarding TA biosynthesis, structural characterization of key biosynthetic enzymes, and evolutionary analyses of biosynthetic pathways, this review comprehensively summarizes the latest developments in the field and discusses future research directions. We first outline the early biosynthetic steps leading from putrescine to the key intermediate tropinone, with particular emphasis on metabolic flux partitioning mediated by ornithine decarboxylase (ODC) and arginine decarboxylase (ADC). We then discuss the molecular basis of stereoselective branch formation during tropane scaffold biosynthesis, focusing on tropinone reductase I/II (TRI/TRII) and methylecgonone reductase (MecgoR). Finally, we comprehensively compare the complete biosynthetic pathways leading to hyoscyamine/scopolamine in the Solanaceae and cocaine in the Erythroxylaceae, highlighting their distinct catalytic strategies and evolutionary trajectories (Figure 2). By integrating evidence from genomics, structural biology, and functional evolutionary studies, we further examine the origin, independent loss, and convergent evolution of tropane alkaloid biosynthesis, with the aim of providing a theoretical basis for the rational design and metabolic engineering of TA biosynthetic systems.

## 2. Tropinone Biosynthesis: From Putrescine to Tropinone

The name tropane alkaloids (TA) is derived from their characteristic tropane ring, a unique bicyclic structural framework that defines this class of natural products. Tropinone serves as the central intermediate in TA biosynthesis, and its formation originates from the primary metabolite putrescine [42,43]. Putrescine is a ubiquitous diamine in plants that not only functions as the key precursor for tropane alkaloid biosynthesis but also plays important roles in plant responses to heavy metal stress [44,45].

**Figure 3:**
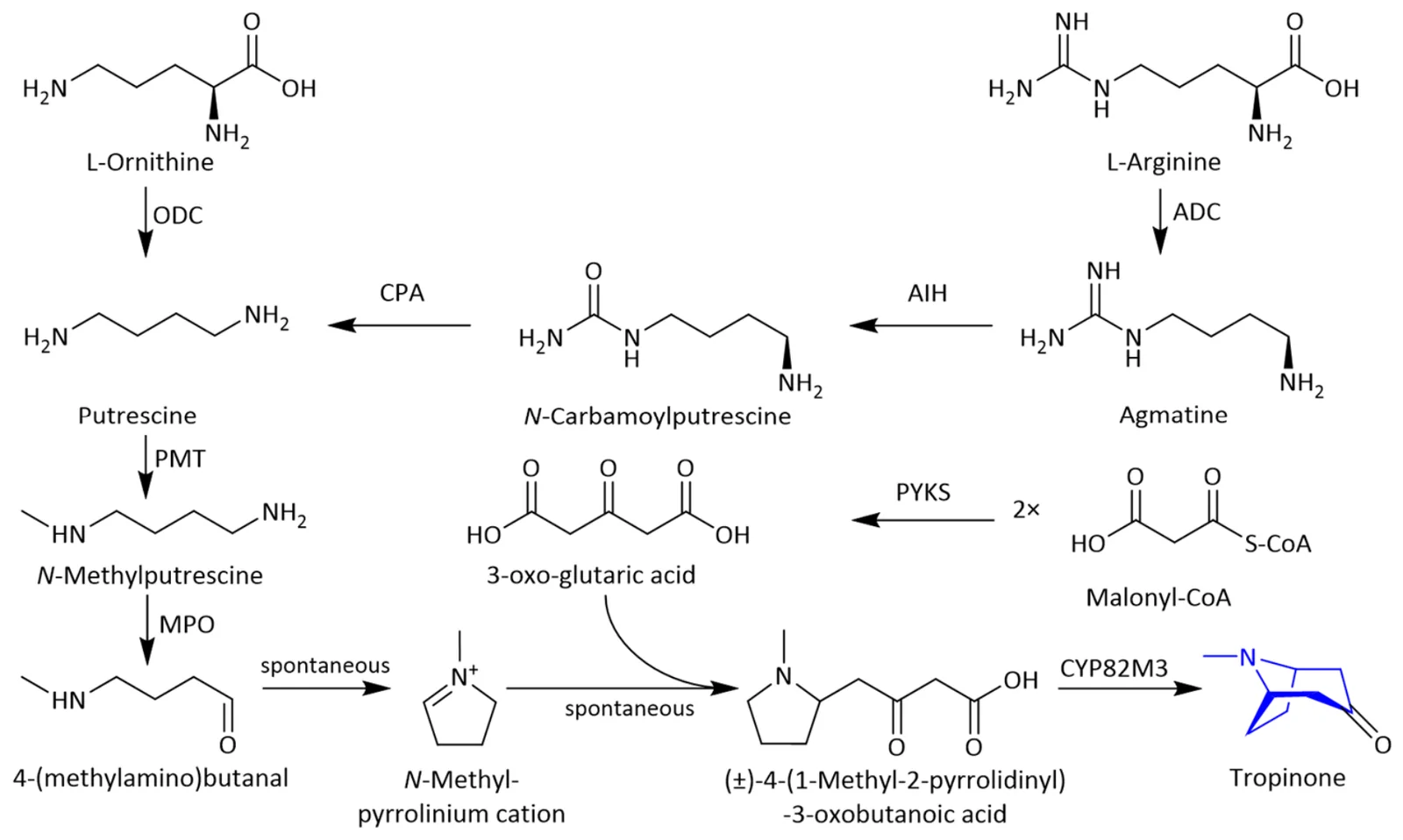
Biosynthetic pathway of tropinone in medicinal solanaceous plants. The blue moiety highlights the conserved tropane bicyclic scaffold common to tropane alkaloids.

In plants, putrescine is synthesized through two parallel pathways: the ornithine decarboxylase (ODC)-mediated ornithine decarboxylation pathway and the arginine decarboxylase (ADC)-mediated arginine decarboxylation pathway. Putrescine *N*-methyltransferase (PMT) catalyzes the methylation of putrescine using S-adenosyl-L-methionine (SAM) as the methyl donor, yielding *N*-methylputrescine. Subsequently, *N*-methylputrescine oxidase (MPO), a copper-dependent diamine oxidase (DAO), catalyzes the oxidative deamination of *N*-methylputrescine to produce 4-(methylamino)butanal, which spontaneously undergoes intramolecular cyclization formation to generate the *N*-methyl-Δ^1^-pyrrolinium cation. Meanwhile, the type III polyketide synthase (PYKS) catalyzes the condensation of two molecules of malonyl-CoA to produce 3-oxoglutaric acid (OGA). This intermediate subsequently undergoes a Mannich condensation with the *N*-methyl-Δ^1^-pyrrolinium cation to form 4-(1-methyl-2-pyrrolidinyl)-3-oxobutanoic acid (MPOA). Finally, tropinone synthase (CYP82M3) catalyzes the oxidative cyclization of racemic 4-(1-methyl-2-pyrrolidinyl)-3-oxobutanoic acid to generate tropinone, the pivotal intermediate from which diverse tropane alkaloids are derived (Figure 3).

### 2.1. Ornithine Decarboxylase-Mediated Putrescine Biosynthesis

Ornithine decarboxylase (ODC) catalyzes the conversion of ornithine to putrescine, a precursor in the biosynthesis of TA and nicotine [46,47]. The ODC-catalyzed step is rate-limiting in the TA biosynthetic pathway, regulating the flux of ornithine into secondary metabolism. ODC genes have been identified in three TA-producing species: *Atropa belladonna*, *Hyoscyamus niger*, and *Anisodus luridus*. Among these, the ODC from *A*. *belladonna* (AbODC) exhibits higher catalytic efficiency than those from *H*. *niger* (HnODC) and *A*. *luridus* (AlODC) [45,46]. AbODC is expressed in all *A*. *belladonna* organs, with the highest expression in lateral roots, where TA biosynthesis occurs. Silencing AbODC decreases the levels of putrescine, *N*-methylputrescine, hyoscyamine, and scopolamine, and causes pollen sterility [46,48]. Conversely, overexpression of AbODC increases putrescine and *N*-methylputrescine levels, resulting in a moderate rise in hyoscyamine and enhanced tolerance to low-temperature stress, attributed to the role of putrescine and its derivatives in stress regulation [49]. Overexpression of a mouse ODC gene in *Datura innoxia* also increases scopolamine content [50].

### 2.2. ADC-Mediated Putrescine Biosynthesis

#### 2.2.1. Arginine Decarboxylase

Arginine decarboxylase (ADC) catalyzes the first and rate-limiting step of the ADC-mediated putrescine biosynthetic pathway, converting L-arginine into agmatine through decarboxylation [51]. Unlike ornithine decarboxylase (ODC), which is generally encoded by a single-copy gene and primarily functions in cell division, plant ADC is typically encoded by a multigene family and plays essential roles in non-dividing cells, tissue elongation, and responses to environmental stresses [52]. From an evolutionary perspective, the ADC pathway exhibits unique significance in plants. For example, the model plant *Arabidopsis thaliana*, several members of the Brassicaceae, and *Spirodela polyrhiza* completely lack functional ODC genes, rendering the ADC pathway the sole route for the biosynthesis of putrescine and downstream polyamines in these species [53,54].

The ADC pathway also plays a pivotal role in TA-producing plants. In hairy root cultures of *Datura stramonium*, inhibition of ADC activity using the specific inhibitor DFMA markedly decreases the levels of free and conjugated putrescine as well as downstream TA intermediates, including *N*-methylputrescine, whereas the ODC inhibitor DFMO exerts only minor effects. These findings indicate that ADC contributes more substantially than ODC to tropane alkaloid biosynthesis in *D*. *stramonium* [55]. In another well-known TA-producing species, *Erythroxylum coca*, the principal alkaloid cocaine also utilizes this pathway to supply its initial precursor, although its tissue specificity differs markedly from that observed in Solanaceous species. Combined ^13^CO_2_ isotope-labeling and quantitative gene expression analyses have demonstrated that TA biosynthesis and EcADC expression occur predominantly in aerial tissues, particularly leaf buds and newly expanded young leaves, rather than in roots. Arginine is highly abundant in *E. coca*, with concentrations exceeding those of ornithine by more than two orders of magnitude, while the transcript level of EcADC is approximately tenfold higher than that of EcODC across all examined tissues [56]. These observations suggest that the ADC-mediated pathway is the predominant route for constitutive cocaine biosynthesis in *E. coca*.

Functional divergence is commonly observed among members of the ADC gene family. In *A*. *belladonna*, six ADC genes have been identified; however, only AbADC1 and AbADC2 are highly expressed in secondary roots, the primary site of TA biosynthesis. RNA interference (RNAi) experiments further demonstrated that suppression of AbADC1 significantly reduces root biomass without substantially affecting TA accumulation, whereas silencing AbADC2 markedly decreases the levels of putrescine, *N*-methylputrescine, hyoscyamine, and scopolamine. These findings indicate that AbADC1 primarily regulates root development, whereas AbADC2 is specifically responsible for directing metabolic flux toward TA biosynthesis [57].

#### 2.2.2. Agmatine Iminohydrolase

Agmatine iminohydrolase (AIH) catalyzes the second step of the ADC pathway by hydrolyzing agmatine, the product of arginine decarboxylation, into *N*-carbamoylputrescine with the concomitant release of ammonia [58]. In plants, AIH exhibits complex subcellular localization patterns that imply sophisticated regulatory mechanisms. In *Arabidopsis thaliana*, five AIH isoforms (AtAIH.1–AtAIH.5) are generated through alternative splicing. Fluorescence localization analyses have shown that most isoforms, including AtAIH.1 and AtAIH.2, are predominantly localized to the endoplasmic reticulum (ER), whereas the unique AtAIH.4 isoform is targeted to chloroplasts owing to its distinctive *N*-terminal targeting sequence. This spatial compartmentalization between the ER and plastids suggests that plants may regulate polyamine metabolic flux within different organelles to accommodate diverse physiological demands and environmental challenges [59]. Studies have further shown that homozygous AIH mutants frequently exhibit severe defects in embryonic development as a consequence of disrupted polyamine metabolism, highlighting the indispensable role of this hydrolytic step in both fundamental plant development and secondary metabolic networks [60]. To date, however, studies investigating AIH genes involved in tropane alkaloid biosynthesis remain scarce, and no direct functional characterization has yet been reported.

#### 2.2.3. *N*-Carbamoylputrescine Amidohydrolase

*N*-carbamoylputrescine amidohydrolase (CPA), a member of the C–N hydrolase superfamily, catalyzes the final step of the ADC-mediated putrescine biosynthetic pathway by specifically hydrolyzing *N*-carbamoylputrescine to produce putrescine and ammonia [61]. In some plants, the ADC pathway serves as the principal route for putrescine biosynthesis. For example, because *Arabidopsis thaliana* lacks a functional ODC gene, putrescine production relies exclusively on this pathway. Structurally and biochemically, CPA typically assembles into an octameric complex and exhibits positive cooperative allosteric behavior toward its substrate. At the subcellular level, CPA is predominantly localized to the ER. This specific compartmentalization effectively excludes the terminal step of putrescine biosynthesis from the cytosol. Since the upstream reactions of the ADC pathway occur partially within chloroplasts, the close spatial association between ER-localized CPA and chloroplasts facilitates efficient channeling of metabolic intermediates. Such an intracellular organization not only promotes intercellular transport and vesicle-mediated secretion of putrescine through the ER network and plasmodesmata but also ensures a continuous supply of this essential precursor for the biosynthesis of downstream specialized metabolites, including tropane alkaloids [59,62]. Similar to AIH, studies focusing on CPA genes involved in tropane alkaloid biosynthesis remain extremely limited, and no direct reports describing their functional roles have yet been published.

### 2.3. Putrescine N-Methyltransferase

Putrescine *N*-methyltransferase (PMT) catalyzes the transfer of a methyl group from *S*-adenosylmethionine (dcSAM) to putrescine, producing *N*-methylputrescine. PMT genes are expressed specifically in lateral roots of *A*. *belladonna* and tobacco, with the encoded proteins localized to the pericycle [63,64]. PMT plays a role upstream in both nicotine and TA biosynthesis, making it valuable for metabolic engineering. Overexpression of PMT in *Nicotiana sylvestris* increases nicotine content [65], but its effects on TA vary across species. In *Hyoscyamus muticus* hairy roots, PMT overexpression moderately increases hyoscyamine content [66], while in *A*. *belladonna* and *H*. *niger* hairy roots, it has no significant effect on hyoscyamine or scopolamine levels [67,68]. Nevertheless, simultaneous expression of PMT and H6H in hairy roots of *A*. *belladonna* and *H*. *niger* markedly enhances scopolamine accumulation compared with H6H overexpression alone [69,70,71]. These findings suggest that PMT exerts species-specific regulatory effects on metabolic flux in TA-producing plants.

### 2.4. N-Methylputrescine Oxidase in Solanaceae

*N*-methylputrescine oxidase (MPO) catalyzes the oxidative deamination of *N*-methylputrescine, producing 4-(methylamino)butanal, which cyclizes into the *N*-methyl-Δ^1^-pyrrolinium cation [72]. This step is the final shared node in the biosynthesis of TA and nicotine. MPO belongs to the diamine oxidase (DAO) family, which uses copper ions and topaquinone as cofactors to catalyze the oxidation of polyamines like putrescine and cadaverine. Phylogenetic analysis reveals that MPO is homologous to typical putrescine-oxidizing DAOs, suggesting its evolution from this subfamily [73]. MPOs have been identified in species such as *A*. *belladonna*, *Datura* spp., *H*. *niger*, and tobacco, with MPO showing a stronger catalytic preference for *N*-methylputrescine over putrescine or cadaverine [26,39,74,75].

In *A*. *belladonna*, two MPO homologs, AbMPO1 and AbMPO2, share high sequence homology with tobacco MPO1 (NtMPO1). AbMPO1 is highly expressed in lateral roots, coinciding with the location of TA biosynthesis, while AbMPO2 is more widely expressed, indicating a limited role in TA biosynthesis. RNA interference (RNAi) silencing of AbMPO1 resulted in significantly reduced hyoscyamine and scopolamine accumulation in *A*. *belladonna* hairy roots, whereas silencing AbMPO2 had no significant effect. These findings confirm that AbMPO1 is the primary MPO involved in TA biosynthesis in *A*. *belladonna* [39].

### 2.5. Type III Polyketide Synthase

Type III polyketide synthases (PKSs), like chalcone synthase, typically catalyze the stepwise elongation of polyketide backbones using CoA-thioesterified molecules. In the TA biosynthetic pathway, PYKS, a specialized type III PKS, mediates the formation of 3-oxoglutaric acid, which produces the key intermediate for tropinone synthesis [76]. PYKS in *A*. *belladonna* was identified using in vitro enzymatic assays and virus-induced gene silencing (VIGS). Silencing PYKS resulted in reduced hyoscyamine and scopolamine content, accompanied by the accumulation of *N*-methyl-Δ^1^-pyrrolinium cation, confirming its involvement in TA biosynthesis [77]. The crystal structure of PYKS was resolved by Huang et al., and its mechanism was further elucidated through protein co-crystallization and enzymatic assays. PYKS catalyzes the condensation of two malonyl-CoA molecules to form 3-oxoglutaric acid, which undergoes nonenzymatic Mannich-like condensation with *N*-methyl-Δ^1^-pyrrolinium cation, producing racemic 4-(1-Methyl-2-pyrrolidinyl)-3-oxobutanoic acid [78].

### 2.6. Tropinone Synthase

Tropinone synthase (CYP82M3) catalyzes the conversion of 4-(1-methyl-2-pyrrolidinyl)-3-oxobutanoic acid to tropinone, a core intermediate in the biosynthesis of medicinal tropane alkaloids (TAs). As a crucial node within the pathway, this step plays an important role in directing metabolic flux toward tropane skeleton formation and thereby influences the accumulation of downstream alkaloids, including scopolamine and hyoscyamine. CYP82M3 was initially isolated from *A*. *belladonna* and its involvement in tropinone biosynthesis was subsequently validated using gene co-expression analysis combined with virus-induced gene silencing (VIGS). Furthermore, reconstruction of the entire biosynthetic pathway in *Nicotiana benthamiana* leaves enabled de novo heterologous production of tropinone, which verified the catalytic activity of AbCYP82M3 and provided a foundation for metabolic engineering strategies aimed at enhancing TA production [77].

## 3. Tropinone Reductases: Stereoselective Branching of Tropane Alkaloid Biosynthesis

Tropinone represents a key metabolic branch point in the TA biosynthetic pathway. It can be stereoselectively reduced by two distinct enzymes to produce either tropine or pseudotropine, thereby directing metabolic flux toward different downstream alkaloid classes.

### 3.1. Tropinone Reductase I

Tropinone reductase I (TRI) catalyzes the NADPH-dependent reduction in tropinone to tropine (3α-hydroxytropane), the indispensable precursor for medicinally important TAs, including hyoscyamine and scopolamine [79,80]. Metabolic engineering studies have demonstrated that overexpression of TRI in hairy roots of *Anisodus acutangulus* significantly enhances the accumulation of tropane alkaloids, particularly scopolamine [81]. Furthermore, co-expression of TRI and H6H (hyoscyamine 6β-hydroxylase) produces an even greater increase in scopolamine production, highlighting the importance of TRI in optimizing carbon flux toward hyoscyamine and scopolamine [82]. These findings establish TRI as an important engineering target for improving hyoscyamine and scopolamine biosynthesis.

### 3.2. Tropinone Reductase II

Tropinone reductase II (TRII) catalyzes the stereospecific reduction in the C3 keto group of tropinone to produce pseudotropine (3β-hydroxytropane) [83,84]. In contrast to TRI, which generates tropine with the 3α-hydroxyl configuration, TRII directs metabolic flux toward the calystegine biosynthetic branch and therefore functions as one of the key branch-point enzymes in TA metabolism [12,85]. Like TRI, TRII belongs to the short-chain dehydrogenase/reductase (SDR) superfamily and catalyzes NADPH-dependent oxidoreduction reactions using NAD(P)(H) as a cofactor. TRII was first purified from the roots of *H*. *niger*. Early biochemical studies demonstrated that pseudotropine does not spontaneously isomerize into tropine, providing compelling evidence that the two stereoisomeric products are generated through two independent enzymatic reactions rather than by post-synthetic interconversion [86]. Consequently, TRI and TRII constitute the principal branch point controlling carbon flux within the TA biosynthetic pathway. Since then, the genes encoding TRI and TRII have been isolated and characterized from numerous Solanaceous species. The two enzymes share more than 50% amino acid sequence identity, suggesting that they originated from gene duplication of a common ancestral SDR enzyme followed by functional divergence. Immunolocalization studies in potato further revealed that Tropinone Reductases are predominantly localized in roots and tubers, consistent with the well-established role of roots as the principal site of TA biosynthesis [87,88,89,90,91].

**Figure 4:**
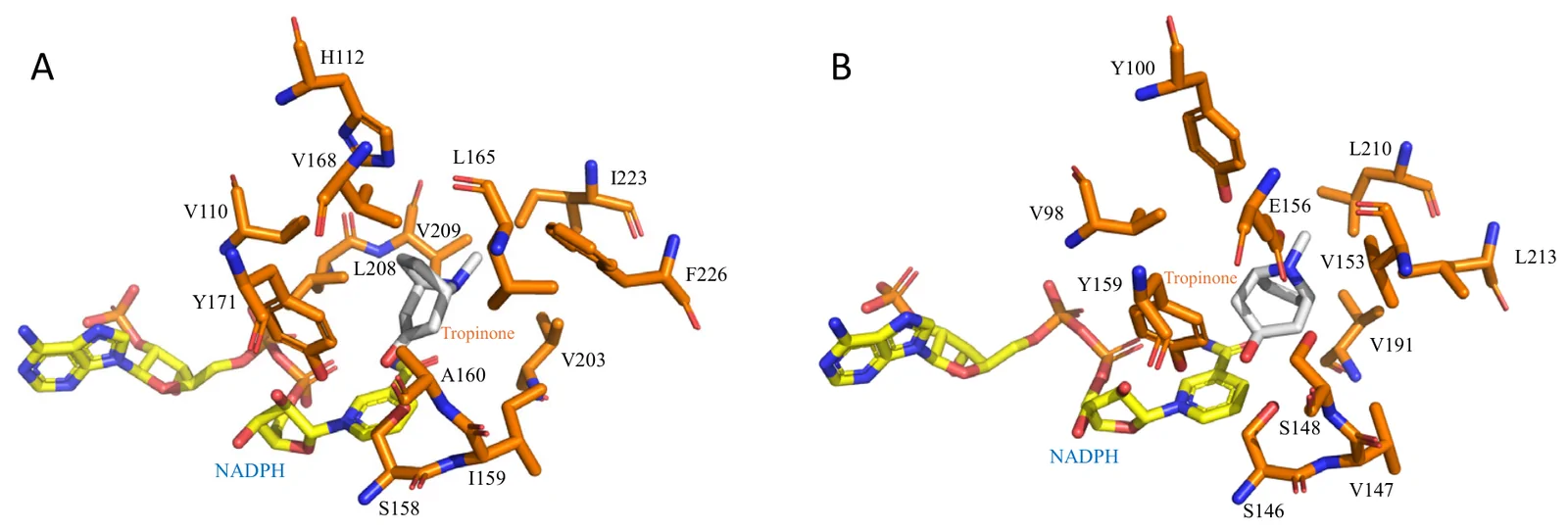
Structural comparison of the tropinone-binding pockets of tropinone reductase I (TRI) and tropinone reductase II (TRII) underlying stereoselective reduction in tropinone. Predicted tropinone-binding pockets of TRI (**A**) and TRII (**B**), respectively, showing tropinone, NADPH, and the key amino acid residues surrounding the substrate-binding sites. Tropinone is shown in gray; NADPH is shown in yellow; nitrogen atoms are colored blue, oxygen atoms are colored red, and the side chains of the key amino acid residues are shown as orange sticks. The structures were visualized using PyMOL 3.0.3.

Crystal structures of TRI and TRII from *D*. *stramonium* revealed that the two enzymes possess nearly identical overall folds and share approximately 64% amino acid sequence identity, further supporting their relatively recent evolutionary divergence from a common ancestral protein. Despite their highly conserved structures, subtle differences in active-site residues determine their opposite stereochemical outcomes. In TRI, a histidine residue introduces a localized positive charge that electrostatically repels the positively charged nitrogen atom of tropinone, thereby altering substrate orientation within the catalytic pocket and favoring reduction to tropine with the 3α-hydroxyl configuration. In TRII, this histidine is replaced by tyrosine, while a glutamate residue occupies the position corresponding to valine in TRI, generating a more negatively charged active-site environment. The predicted tropinone-binding pockets of TRI and TRII, together with their key active-site residues, are shown in Figure 4.

These electrostatic differences dictate substrate binding orientation and ultimately determine product stereochemistry. Remarkably, substitution of little amino acid residues is sufficient to switch the stereospecificity between TRI and TRII, illustrating how minor structural modifications can profoundly alter enzyme function. Furthermore, biochemical characterization in *H*. *niger* demonstrated that TRI catalyzes a reversible reaction, whereas TRII functions exclusively as a unidirectional reductase [92,93].

From the perspective of metabolic engineering, the tropinone branch point controlled by TRI and TRII represents one of the most promising regulatory nodes for manipulating TA biosynthesis. TRI channels metabolic intermediates toward the production of hyoscyamine and scopolamine, whereas TRII diverts carbon flux into the calystegine pathway. Therefore, increasing the relative expression or activity of TRI, or suppressing TRII through RNA interference (RNAi) or gene knockout, is expected to redirect a larger proportion of metabolic flux toward the biosynthesis of pharmaceutically valuable tropane alkaloids. In *Anisodus acutangulus*, overexpression of TRI has already been shown to significantly increase hyoscyamine and scopolamine accumulation in hairy roots [81]. Combining TRI overexpression with targeted suppression of TRII may therefore constitute an effective metabolic engineering strategy for enhancing the production of these medically important alkaloids.

Although TRI and TRII represent well-characterized examples of enzyme-driven stereochemical diversification, several questions remain unresolved. Most biochemical studies have focused on a limited number of Solanaceous species, and the extent to which TRI/TRII homologs from other lineages exhibit similar catalytic properties remains unclear. In addition, metabolic engineering strategies targeting this branch point have mainly been evaluated in plant hairy root systems, whereas their effectiveness in microbial hosts requires further investigation.

## 4. Biosynthesis of Hyoscyamine and Scopolamine from Tropine

**Figure 5:**
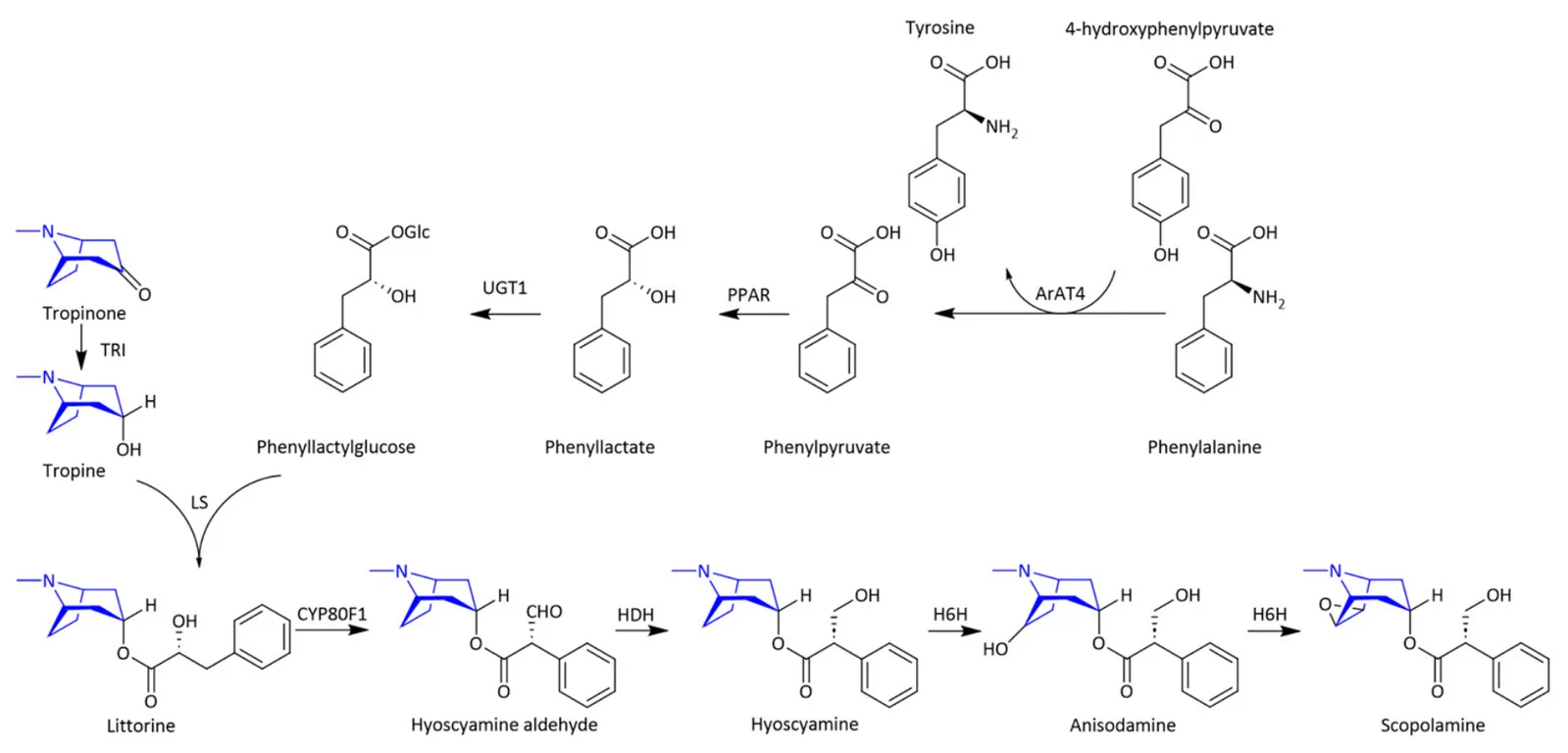
Biosynthetic pathway of hyoscyamine and scopolamine originating from tropinone.

The biosynthesis of hyoscyamine originates from phenylalanine-derived phenyllactylglucose and proceeds via littorine, rather than through the direct condensation of tropine and tropic acid [94]. Tropinone undergoes initial reduction to generate tropine under the catalysis of TRI, and this compound functions as a crucial acyl acceptor during tropane alkaloid biosynthesis. Phenylalanine metabolic processes contribute to the formation of the tropic acid moiety within hyoscyamine. Through sequential catalysis by *L-phenylalanine*: 4-Hydroxyphenylpyruvate Aminotransferase 4 (ArAT4), Phenylpyruvic Acid Reductase (PPAR), and Phenyllactate UDP-glycosyltransferase (UGT1), phenylalanine is converted to phenyllactylglucose, which then participates in the biosynthesis of littorine, a precursor of hyoscyamine and scopolamine. Littorine, a key intermediate in hyoscyamine biosynthesis, undergoes intramolecular rearrangement catalyzed by littorine mutase (CYP80F1) to form hyoscyamine aldehyde. This intermediate is subsequently reduced to hyoscyamine by hyoscyamine dehydrogenase (HDH). In the scopolamine biosynthetic pathway, hyoscyamine 6β-hydroxylase (H6H) catalyzes the 6β-hydroxylation of hyoscyamine to anisodamine, which is then epoxidized to scopolamine, completing the final maturation of medicinal tropane alkaloids (Figure 5).

### 4.1. L-Phenylalanine: 4-Hydroxyphenylpyruvate Aminotransferase 4

*L-phenylalanine*: 4-Hydroxyphenylpyruvate Aminotransferase 4 (ArAT4) mediates the transamination reaction between phenylalanine and 4-hydroxyphenylpyruvate, leading to the formation of phenylpyruvic acid—a direct precursor of phenyllactate. ArAT4 is of crucial importance in bridging the primary metabolism of aromatic amino acids and the secondary metabolism of tropane alkaloids. The Ab-ArAT4 gene, identified in *A*. *belladonna*, is highly expressed in root tissues, consistent with the site of TA biosynthesis. Functional assays show that Ab-ArAT4 preferentially catalyzes the formation of phenylpyruvic acid, demonstrating its role in regulating TA precursor supply. Silencing Ab-ArAT4 reduces phenyllactate, hyoscyamine, and scopolamine content by 95%, 77%, and 66%, respectively, while upstream substrates remain unchanged, indicating its critical role in regulating TA biosynthesis [95].

### 4.2. PPhenylpyruvic Acid Reductase

Phenylpyruvic Acid Reductase (PPAR) facilitates the conversion of phenylpyruvic acid into phenyllactate via a reductive reaction. The resultant phenyllactate acts as a vital precursor for littorine formation, and littorine is an indispensable intermediate involved in the biosynthetic pathway of TA. PPAR plays a critical role in linking phenylalanine metabolism to TA production by regulating the flux of precursors required for downstream TA synthesis, including hyoscyamine and scopolamine. The PPAR gene in *A*. *belladonna* is expressed in lateral roots, primarily in pericycle and endodermal cells, aligning with the site of TA biosynthesis. PPAR demonstrates substrate promiscuity, efficiently reducing phenylpyruvic acid to phenyllactate, while its catalytic activity toward p-hydroxyphenylpyruvate is much weaker. Silencing PPAR significantly reduces phenyllactate accumulation, along with decreases in hyoscyamine, anisodamine, and scopolamine levels, confirming the critical role of phenyllactate in TA biosynthesis [96].

### 4.3. Phenyllactate UDP-Glycosyltransferase

Phenyllactate UDP-glycosyltransferase (UGT1) catalyzes the glycosylation of phenyllactate to form phenyllactylglucose, a key acyl donor for littorine biosynthesis. This glycosylation step is crucial for activating phenyllactate in the biosynthetic pathway, replacing the previously hypothesized CoA ligation mechanism. In *A*. *belladonna*, the expression of UGT1 is predominantly localized to lateral roots, which coincides with the primary tissue where TA biosynthesis occurs. Functional validation through gene silencing and overexpression has confirmed that UGT1 plays a central role in TA production. Silencing UGT1 results in reduced accumulation of littorine, hyoscyamine, and scopolamine, while overexpression increases the levels of these compounds, demonstrating UGT1’s role in optimizing metabolic flux and enhancing TA biosynthesis [97].

### 4.4. Littorine Synthase

Littorine synthase (LS) catalyzes the esterification of phenyllactylglucose with tropine to form littorine, a critical intermediate in the biosynthesis of hyoscyamine and scopolamine. This step drives metabolic flux toward hyoscyamine and scopolamine from precursor intermediates. Earlier studies suggested that phenyllactyl-CoA, formed by CoA ligase, acts as the acyl donor for littorine synthesis [98]. However, the discovery of UGT1 refuted this, proposing that serine carboxypeptidase-like (SCPL) acyltransferases are key for littorine synthesis. LS, identified in *A*. *belladonna*, is expressed in lateral roots, correlating with TA biosynthesis. Silencing LS significantly reduces littorine, hyoscyamine, and scopolamine levels, while overexpression increases their accumulation, further enhanced by co-expression with UGT1. LS’s function was validated through the de novo synthesis of littorine in yeast, confirming its role in the biosynthetic pathway and providing a critical enzyme for efficient TA production [97].

### 4.5. Littorine Mutase

Littorine mutase (CYP80F1), a cytochrome P450 enzyme, catalyzes the conversion of littorine to hyoscyamine aldehyde via intramolecular rearrangement. This step is essential for forming the tropic acid skeleton and serves as a structural foundation for hyoscyamine biosynthesis. CYP80F1, first identified in *H*. *niger*, is expressed in roots, consistent with the primary site of TA biosynthesis. Silencing CYP80F1 significantly blocks hyoscyamine accumulation, causing a build-up of littorine. Microsomal fractions from yeast expressing CYP80F1 catalyze this conversion, confirming its role in the rearrangement process. In tobacco hairy roots overexpressing CYP80F1, hyoscyamine is produced when fed littorine, further validating its function in the biosynthesis pathway [99]. Studies on scopolamine synthesis in yeast reinforce CYP80F1’s essential role in the post-modification module of tropane alkaloid biosynthesis [27].

### 4.6. Hyoscyamine Dehydrogenase

Hyoscyamine dehydrogenase (HDH), an alcohol dehydrogenase, catalyzes the reduction in hyoscyamine aldehyde to hyoscyamine, a crucial step in the terminal biosynthesis of medicinal TAs. HDH participates in the synthetic process of hyoscyamine and scopolamine as well. Overexpression of HDH in *A*. *belladonna* hairy roots significantly increases hyoscyamine and scopolamine content, highlighting its role in enhancing the metabolic flux toward terminal products. Silencing HDH results in the accumulation of hyoscyamine aldehyde without a corresponding decrease in hyoscyamine or scopolamine, indicating that other reductases may compensate for its loss. HDH’s catalytic function and its role in regulating metabolic flux were further validated through in vitro assays and structural analysis. This study emphasizes HDH as a key regulatory enzyme in the metabolic engineering of TAs, offering a target for optimizing hyoscyamine and scopolamine production [27,33].

### 4.7. Hyoscyamine 6β-Hydroxylase

Hyoscyamine 6β-hydroxylase (H6H) catalyzes the 6β-hydroxylation of hyoscyamine to form anisodamine, which is subsequently epoxidized to scopolamine, completing the final structural maturation of TA. As a non-heme iron monooxygenase, H6H requires cofactors such as Fe^2+^, α-ketoglutarate (2-oxoglutaric acid), and O_2_ to carry out these reactions, which involve oxidative hydroxylation [34]. H6H is important for the bioconversion of hyoscyamine to scopolamine, with its activity directly influencing scopolamine levels. Overexpression of H6H increases scopolamine content by 2–5 times in various plant species, while its knockout completely abolishes scopolamine production in *A*. *belladonna*. These findings emphasize H6H’s role as a metabolic valve regulating the distribution of TA and its importance in metabolic engineering and synthetic biology for high-yield scopolamine production [100,101].

Despite substantial progress in elucidating the pathway toward hyoscyamine and scopolamine, several enzymatic steps require further investigation. The activities of core enzymes such as CYP80F1 and H6H have been supported by biochemical characterization and heterologous reconstruction, whereas regulatory mechanisms controlling precursor supply, enzyme compartmentalization, and metabolic channeling remain less well understood. In particular, improving pathway efficiency will require a better understanding of intracellular transport and the interaction between primary metabolism and specialized metabolite biosynthesis.

## 5. Biosynthesis of Pseudotropine-Derived Tropane Alkaloids

Tropane alkaloids containing the pseudotropine stereochemical scaffold are mainly represented by two major groups. One comprises the calystegines, which are widely distributed among diverse plant species, whereas the other consists of cocaine, a characteristic tropane alkaloid produced exclusively by members of the Erythroxylaceae. Calystegine Biosynthesis originates from tropinone, which is stereoselectively reduced by TRII to pseudotropine, followed by a series of enzymatic modifications that ultimately generate different calystegine derivatives. Depending on the number, position, and stereochemistry of hydroxyl groups, these compounds are classified into calystegines A, B, and C. Although cocaine also contains the pseudotropine skeleton, elucidation of its complete biosynthetic pathway has revealed that each biosynthetic step is catalyzed by enzymes distinct from those involved in tropane alkaloid biosynthesis in Solanaceous species. The evolutionary origin of cocaine biosynthesis therefore represents a classic example of convergent evolution in plant specialized metabolism.

### 5.1. Calystegine Biosynthesis

#### 5.1.1. 3β-Tigloyloxytropane Synthase

Structurally, calystegines can be regarded as products derived from pseudotropine through *N*-demethylation and multiple hydroxylation reactions. However, recent studies have demonstrated that Calystegine Biosynthesisdoes not proceed through direct modification of pseudotropine. Instead, pseudotropine first undergoes acylation, predominantly tigloylation or acetylation, to generate *O*-acyl pseudotropine esters, which subsequently serve as substrates for *N*-demethylation and hydroxylation. 3β-Tigloyloxytropane synthase (TS) is the first pathway-specific and rate-limiting enzyme committed to the calystegine branch. TS belongs to the BAHD acyltransferase superfamily. Unlike most BAHD acyltransferases, which are localized in the cytosol, the *A*. *belladonna* enzyme AbTS contains a mitochondrial transit peptide and represents the first mitochondrial-localized BAHD acyltransferase reported to date. This unique subcellular localization effectively separates the acylation reactions of pseudotropine from those of tropine, thereby maintaining metabolic specificity between the two competing TA branches [102].

In vitro kinetic analyses using recombinant AbTS demonstrated that the enzyme preferentially utilizes pseudotropine (3β-tropanol) and tigloyl-CoA to produce 3β-tigloyloxytropane. In contrast, AbTS exhibits only negligible affinity toward benzoyl-CoA or acetyl-CoA. Structural analyses revealed that Phe46 within the substrate-binding pocket generates steric hindrance that prevents aromatic acyl-CoA substrates from accessing the catalytic center, thereby conferring strong specificity for short-chain unsaturated acyl-CoA donors. Substitution of Phe46 with isoleucine partially restored catalytic activity toward benzoyl-CoA, confirming that this residue is a critical determinant of acyl-donor selectivity. Virus-induced gene silencing (VIGS) of AbTS in *A*. *belladonna* seedlings resulted in substantial reductions in the accumulation of 3β-tigloyloxytropane, acetylpseudotropine, multiple hydroxylated *O*-acyl pseudotropine intermediates, and the final product calystegine A3, whereas pseudotropine accumulated significantly. In contrast, the contents of littorine, hyoscyamine, and scopolamine remained unchanged, directly demonstrating that TS specifically regulates metabolic flux through the pseudotropine branch without affecting the tropine-derived biosynthetic pathway. Conversely, overexpression of AbTS significantly increased the accumulation of both 3β-tigloyloxytropane and acetylpseudotropine in *A*. *belladonna* hairy roots, further confirming its central role in Calystegine Biosynthesis [102].

#### 5.1.2. CYP450-Mediated Biosynthesis of Calystegines A3, A5, and B1

**Figure 6:**
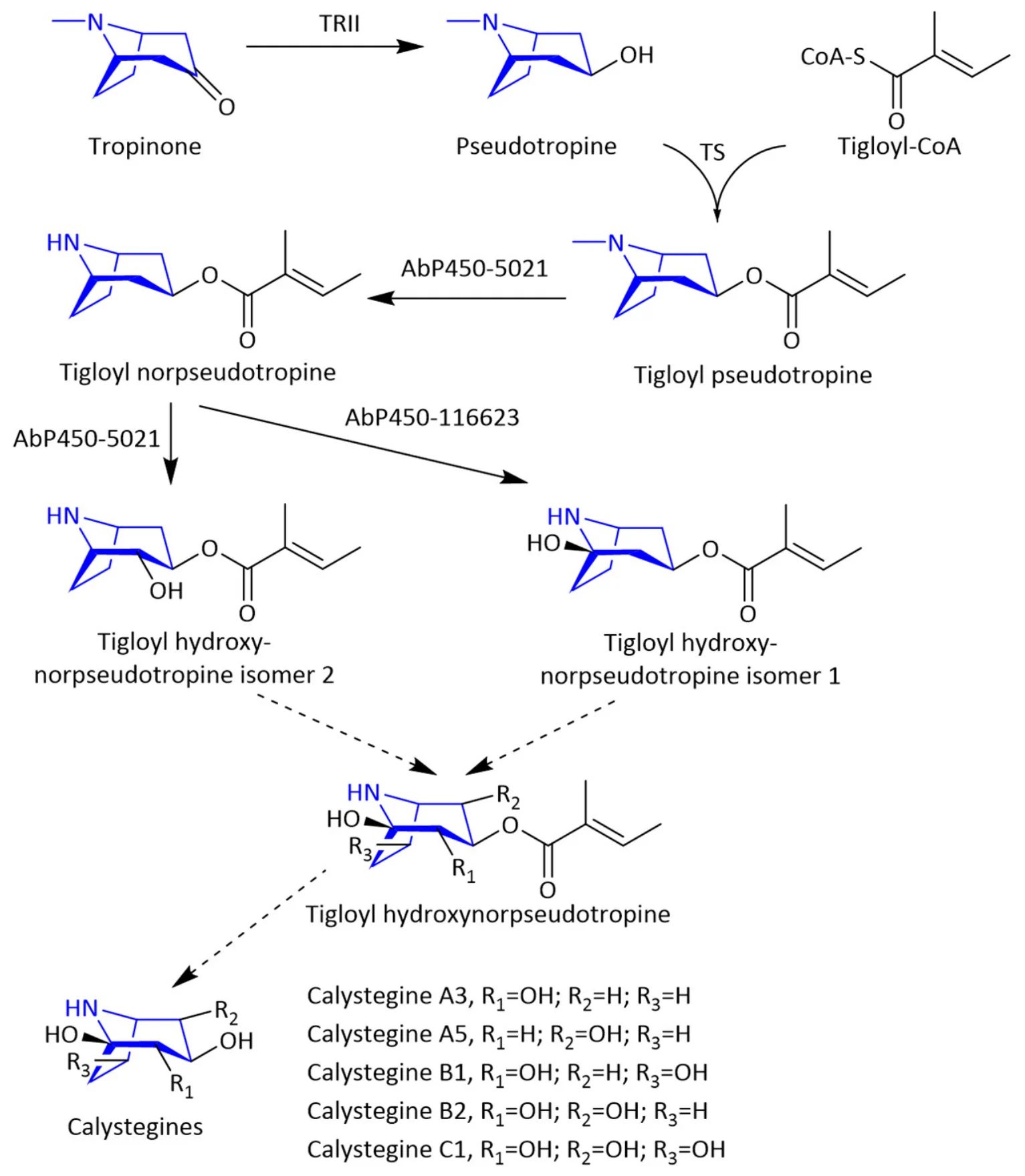
Biosynthetic pathway of calystegines originating from tropinone. Dashed arrows indicate unidentified genes or enzymes; multiple enzymes may be involved in the corresponding catalytic steps.

Following the formation of 3β-tigloyloxytropane, Calystegine Biosynthesis proceeds through cytochrome P450-mediated *N*-demethylation and hydroxylation reactions, followed by hydrolysis to generate the corresponding calystegines. In *A*. *belladonna*, two functionally differentiated cytochrome P450 enzymes, AbP450-5021 (CYP71 family) and AbP450-116623 (CYP82 family), catalyze the sequential oxidative modifications of 3β-tigloyloxytropane. Integrated metabolomic analyses combined with VIGS demonstrated that both P450 enzymes specifically participate in pseudotropine-derived metabolism and regulate the biosynthesis of calystegines A3, A5, and B1. Silencing either gene significantly reduced the accumulation of these calystegines while causing substantial accumulation of upstream intermediates, including pseudotropine and 3β-tigloyloxytropane. In contrast, hyoscyamine and scopolamine levels remained unaffected, confirming that these two CYP450 enzymes function exclusively within the calystegine branch and are not involved in the biosynthesis of tropine-derived alkaloids. Substrate specificity analyses further revealed distinct catalytic properties for the two enzymes. AbP450-5021 exhibits bifunctional activity, catalyzing both the *N*-demethylation of 3β-tigloyloxytropane and the subsequent hydroxylation of *N*-demethylated 3β-tigloyloxytropane. In comparison, AbP450-116623 specifically catalyzes the hydroxylation of *N*-demethylated *O*-acyl pseudotropines [103]. Although the upstream pathway leading to calystegine formation has now been largely elucidated, the downstream hydroxylases and hydrolytic enzymes responsible for completing Calystegine Biosynthesis remain unidentified and require further investigation (Figure 6).

### 5.2. Cocaine Biosynthesis

Cocaine is the characteristic tropane alkaloid produced by species of the genus Erythroxylum and has long been used clinically as a local anesthetic. Although its molecular structure contains the characteristic 3β-hydroxytropane (pseudotropine) scaffold, early studies assumed that its tropane skeleton was synthesized through essentially the same pathway as that of Solanaceous tropane alkaloids. Recent biochemical and evolutionary studies, however, have demonstrated that cocaine biosynthesis represents a striking example of convergent evolution. Despite producing a structurally similar tropane scaffold, plants of the family Erythroxylaceae employ an entirely distinct set of enzymes and biosynthetic strategies from those utilized by Solanaceae.

#### 5.2.1. Spermidine Synthase/*N*-Methyltransferase

Spermidine Synthase/*N*-methyltransferase (EnSPMT1) belongs to the spermidine synthase (SPDS) family and represents a unique bifunctional enzyme that combines spermidine synthase and spermidine *N*-methyltransferase (SMT) activities. It catalyzes the first committed step of the polyamine branch leading to cocaine biosynthesis by generating the methylated precursor required for downstream metabolism. Unlike Solanaceous plants such as *A*. *belladonna*, which recruit an independently evolved putrescine *N*-methyltransferase (PMT) to directly methylate putrescine, EnSPMT1 exhibits dual catalytic activities. Using decarboxylated S-adenosylmethionine (dcSAM), it first catalyzes the canonical aminopropyl transfer reaction shared by canonical plant spermidine synthases (SPDS), which exclusively convert putrescine to spermidine without secondary *N*-methyl modification on polyamine skeletons. It subsequently functions as a highly specialized spermidine *N*-methyltransferase (SMT, the universal single-function methyltransferase targeting free polyamines in most angiosperms), utilizing SAM as the methyl donor to specifically methylate the internal N4 atom of spermidine, thereby producing *N*-methylspermidine. This unusual dual-cofactor utilization, involving both SAM and dcSAM, establishes a biosynthetic strategy fundamentally distinct from that employed by Solanaceous species and provides genomic evidence supporting the independent convergent evolution of cocaine biosynthesis [41,104].

#### 5.2.2. FAD-Dependent Amine Oxidase

FAD-Dependent Amine Oxidase (EnAOF1) belongs to the flavin adenine dinucleotide (FAD)-dependent amine oxidase family and functions as the key oxidative cleavage enzyme within the methylated polyamine pathway. It catalyzes the oxidative degradation of *N*-methylspermidine to generate *N*-methylputrescine, the same precursor of TA biosynthesis. Phylogenetically, EnAOF1 evolved from the plant polyamine oxidase (PAO) family and forms a lineage-specific clade unique to Erythroxylaceae together with homologs most closely related to spermidine oxidases from *Arabidopsis thaliana* and *Zea mays*. Whereas canonical plant PAOs generally oxidize secondary amines, EnAOF1 has acquired a novel catalytic function by specifically recognizing the tertiary amine substrate *N*-methylspermidine. Oxidation occurs at the exo carbon adjacent to the methylated N4 atom, resulting in aminopropyl cleavage and formation of *N*-methylputrescine together with 3-aminopropanal. Transient expression of EnAOF1 in tobacco leaves efficiently converted *N*-methylspermidine into *N*-methylputrescine, confirming its physiological function. In contrast, the common ancestor of Solanaceae lost the ancestral AOF1 gene early during evolution, precluding recruitment of this oxidative pathway. Consequently, Solanaceous plants evolved a much shorter pathway by recruiting PMT to directly synthesize *N*-methylputrescine [41,104].

#### 5.2.3. *N*-Methylputrescine Oxidase

*N*-Methylputrescine oxidase 1 (EnMPO1) belongs to the copper-dependent amine oxidase (CuAO) superfamily and typically contains a C-terminal peroxisomal targeting signal, resulting in exclusive localization to plant peroxisomes. EnMPO1, together with its closely related homologs EcAOC1 and EcAOC2, catalyzes the oxidative deamination of the terminal primary amino group of *N*-methylputrescine to produce 4-(methylamino)butanal. This unstable aldehyde subsequently undergoes spontaneous intramolecular cyclization to generate the *N*-methyl-Δ^1^-pyrrolinium cation, a pivotal activated intermediate in cocaine biosynthesis. Although EnMPO1 catalyzes essentially the same biochemical transformation as the *N*-methylputrescine oxidases of Solanaceous species, phylogenetic analyses indicate that the enzymes originated independently. EnMPO1 is more closely related to ancestral diamine oxidases (DAOs) from dicotyledonous plants outside Solanaceae, including castor bean and grapevine, than to Solanaceous MPOs. These findings demonstrate that *N*-methylputrescine oxidase activity arose through parallel recruitment of different ancestral copper amine oxidases in the two plant lineages, providing another compelling example of convergent evolution [41,104].

#### 5.2.4. Type III Polyketide Synthases

EnPKS1 and EnPKS2 are members of the plant type III polyketide synthase (PKS) family and catalyze the key step responsible for constructing the carbon skeleton of the tropane ring precursor. Using two molecules of malonyl-CoA as substrates, these enzymes perform only a single condensation cycle to generate 3-oxoglutaric acid, subsequently undergoes spontaneous, non-enzymatic Mannich condensation with the *N*-methyl-Δ^1^-pyrrolinium cation to produce 4-(1-methyl-2-pyrrolidinyl)-3-oxobutanoic acid, the immediate precursor of tropane ring formation. Structural analyses revealed that EnPKS1/2 functions as a homodimer possessing a highly unusual catalytic pocket formed jointly by residues R212, F216, I255, and S339 from one monomer together with Lys138 contributed by the adjacent monomer. Lys138 plays a critical role by cooperating with Arg212 to stabilize the carboxylate intermediate through hydrogen bonding and salt-bridge interactions, thereby generating steric constraints that terminate chain elongation after a single condensation cycle. Site-directed mutagenesis further demonstrated remarkable catalytic plasticity: substitution of Lys138 with methionine or glutamate abolished 3-oxoglutaric acid (OGA) formation and instead permitted two condensation cycles, yielding the longer-chain product triacetic acid lactone [105].

Remarkably, EnPKS1/2 catalyzes exactly the same chemical transformation as pyrrolidine ketide synthase (PYKS) from Solanaceous species, such as *Anisodus acutangulus*, despite having evolved independently. Whereas Solanaceous PYKS restricts chain elongation through a conserved Arg134 residue, the corresponding position in EnPKS1/2 is occupied by the non-conserved Thr133. Phylogenetic analyses further demonstrate that EnPKS1/2 (Neo-PYKS clade) and Solanaceous PYKS originated from distinct ancestral PKS genes, and their catalytic determinants are not interchangeable. These findings indicate that Erythroxylaceae and Solanaceae independently recruited different ancestral PKS families to fulfill the same biochemical function during tropane alkaloid evolution [78,105].

#### 5.2.5. EnCYP81AN15

EnCYP81AN15 is a lineage-specific cytochrome P450 belonging to the CYP81 family and catalyzes the oxidative cyclization step responsible for formation of the cocaine tropane skeleton. Although this reaction is chemically analogous to that catalyzed by the Solanaceous tropinone synthase CYP82M3, the two enzymes differ fundamentally in both evolutionary origin and catalytic mechanism, making EnCYP81AN15 one of the strongest molecular examples supporting convergent evolution of tropane alkaloid biosynthesis. EnCYP81AN15 selectively catalyzes the oxidative cyclization of the (S)-enantiomer of 4-(1-methyl-2-pyrrolidinyl)-3-oxobutanoic acid to generate the unstable intermediate ecgonone. Functional characterization of EnCYP81AN15 corrected the long-standing hypothesis proposing that methylation precedes cyclization, demonstrating instead that oxidative cyclization occurs before methyl ester formation. The enzyme exhibits strict stereoselectivity, producing exclusively (+)-ecgonone from the (S) substrate. In the absence of rapid downstream methylation by EnMT4, the unstable β-keto acid (+)-ecgonone undergoes spontaneous non-enzymatic decarboxylation to yield achiral tropinone, explaining the substantial accumulation of tropinone observed when EnCYP81AN15 is expressed alone. Sequence comparisons further reveal that EnCYP81AN15 is only distantly related to the Solanaceous CYP82M3, providing additional molecular evidence that cocaine and Solanaceous tropane alkaloids evolved through independent biosynthetic routes [104,106].

#### 5.2.6. Ecgonone Methyltransferase 4

Ecgonone methyltransferase 4 (EnMT4) catalyzes the methyl esterification step linking tropane ring formation with downstream cocaine biosynthesis. For many years, the temporal order of oxidative cyclization and methyl esterification remained controversial. Recent enzymatic studies have unequivocally established that tropane ring formation precedes methylation. EnMT4 specifically methylates the carboxyl group of ecgonone to generate methylecgonone, which subsequently serves as the direct substrate for methylecgonone reductase (MecgoR). The enzyme displays exceptionally stringent substrate specificity and stereoselectivity, efficiently methylating only (+)-ecgonone while exhibiting no detectable activity toward the (−)-enantiomer. This stereochemical discrimination provides a molecular explanation for the exclusive production of a single natural cocaine stereoisomer. Because ecgonone is highly unstable in aqueous solution and readily undergoes spontaneous decarboxylation to form tropinone, rapid methylation by EnMT4 effectively competes with this non-enzymatic side reaction, thereby preserving metabolic flux toward cocaine biosynthesis. Furthermore, biochemical analyses demonstrated that the downstream reductase MecgoR cannot utilize ecgonone directly but specifically recognizes methylecgonone, emphasizing the coordinated metabolic coupling among EnCYP81AN15, EnMT4, and MecgoR [104,106].

#### 5.2.7. Methylecgonone Reductase

Methylecgonone reductase (MecgoR) belongs to the aldo-keto reductase (AKR) superfamily and catalyzes the NADPH-dependent stereospecific reduction in methylecgonone to methylecgonine (2-carbomethoxy-3β-tropine), thereby generating the immediate precursor for the final acylation step catalyzed by cocaine synthase. Although MecgoR is capable of reducing tropinone to pseudotropine in vitro and can catalyze the reverse oxidation reaction, its catalytic efficiency is substantially higher toward methylecgonone owing to favorable interactions with the carbomethoxy substituent. MecgoR also displays pronounced substrate inhibition under physiological conditions. While the enzyme can utilize both NADPH and NADH as hydride donors, catalytic activity is markedly greater with NADPH. Unlike the Solanaceous Tropinone Reductases TRI and TRII, which belong to the short-chain dehydrogenase/reductase (SDR) family, MecgoR shares less than 10% amino acid sequence identity with these enzymes and instead exhibits strong homology with AKR family members such as codeinone reductase involved in morphinan alkaloid biosynthesis. These profound sequence differences indicate that Erythroxylaceae and Solanaceae independently recruited entirely different ancestral enzyme families to catalyze the same C3 reduction in the tropane skeleton [107].

#### 5.2.8. Cocaine Synthase

**Figure 7:**
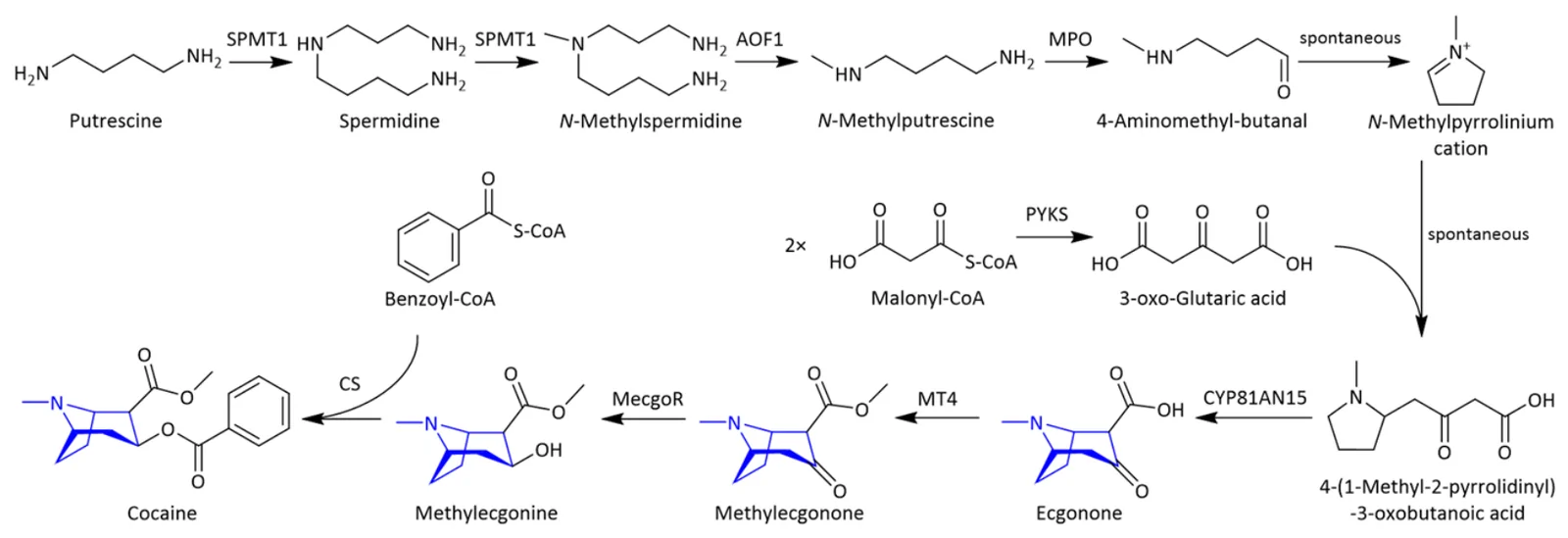
Biosynthetic pathway of cocaine in Erythroxylum coca.

Cocaine synthase (CS) is a member of the plant BAHD acyltransferase family and catalyzes the final step of cocaine biosynthesis by transferring either a benzoyl or cinnamoyl group from the corresponding CoA thioester to the C3 hydroxyl group of methylecgonine (2-carbomethoxy-3β-tropine). This reaction yields cocaine or cinnamoylcocaine, respectively. CS exhibits strict stereochemical specificity toward the acyl acceptor, recognizing only substrates possessing the 3β-hydroxytropane configuration, such as methylecgonine or pseudotropine. In contrast, the enzyme displays broader specificity toward acyl donors, efficiently utilizing both benzoyl-CoA and cinnamoyl-CoA [108]. Owing to its broad substrate scope, CS has become an attractive tool for metabolic engineering aimed at heterologous production of valuable tropane alkaloid derivatives. Heterologous expression of codon-optimized CS in *Saccharomyces cerevisiae*, together with *Arabidopsis* 4-coumarate ligase 5 (At4CL5), enabled microbial biosynthesis of tropane ester derivatives, including benzoyl-3β-tropine and cinnamoyl-3β-tropine, following feeding with benzoic acid or cinnamic acid and pseudotropine [109]. Unlike the root-specific expression pattern characteristic of the Solanaceous TA pathway, CS is predominantly expressed in young expanding leaves, particularly within palisade and spongy mesophyll tissues [40]. This striking spatial divergence further supports the hypothesis that tropane alkaloid biosynthesis originated independently in Erythroxylaceae and Solanaceae (Figure 7).

## 6. Evolution of Tropane Alkaloid Biosynthetic Pathways

Tropane alkaloids exhibit a remarkably discontinuous phylogenetic distribution across angiosperms, occurring sporadically in evolutionarily distant lineages spanning at least seven orders, including the Solanaceae (asterids), Erythroxylaceae (rosids), Convolvulaceae, and Rhizophoraceae. This highly fragmented pattern of chemotypic specialization has long fueled two competing evolutionary hypotheses. The first, the independent-loss model, proposes that the TA pathway originated in an ancient common ancestor of angiosperms and was subsequently lost repeatedly during species diversification. The second, the convergent-evolution model, argues that distantly related plant lineages independently evolved TA biosynthesis by recruiting distinct enzymatic machineries under similar ecological selective pressures. Recent advances in chromosome-scale genome assemblies, comparative genomics, and structural biology have largely reconciled these competing hypotheses. Current evidence indicates that the patchy distribution of medicinal tropane alkaloids within Solanaceae is primarily explained by repeated lineage-specific gene losses following establishment of an ancestral pathway, whereas the biosynthesis of the tropane skeleton in Solanaceae and Erythroxylaceae, which diverged approximately 129 million years ago, represents one of the best-characterized examples of biochemical convergent evolution through recruitment of non-homologous enzymes [39,41].

### 6.1. Evolutionary Loss of Tropane Alkaloid Biosynthesis in Solanaceae

Tropane alkaloids, including hyoscyamine and scopolamine, are restricted to only a few Solanaceous lineages, such as the tribes Hyoscyameae and Datureae, and are absent from most economically important crops, including tomato, potato, and pepper. Comparative evolutionary analyses indicate that the molecular framework of the complete TA biosynthetic pathway had already been established in the common ancestor of Solanaceae. Following the ancient whole-genome triplication (α-WGT) shared by all extant Solanaceous species, an ancestral biosynthetic gene cluster containing the cyclization enzyme CYP82M3 and the reductase TRI was assembled through physical linkage. Subsequently, TRI underwent extensive tandem duplication and lineage-specific expansion, followed by subfunctionalization that ultimately resulted in highly specific expression within roots, particularly secondary roots. This coordinated expansion of the ancestral gene cluster and remodeling of its transcriptional regulation substantially enhanced precursor flux and represented a pivotal event in the establishment of medicinal tropane alkaloid biosynthesis [32,39].

During subsequent species radiation, however, changing ecological conditions, niche transitions, and the considerable metabolic cost associated with producing highly toxic specialized metabolites imposed relaxed selective pressure on the pathway in lineages that no longer required TA production. Consequently, biosynthetic genes accumulated independent degenerative mutations and were progressively lost in multiple Solanaceous crops. Microsynteny analyses combined with comparative genomic sequencing have revealed numerous “genetic fossils” of TA biosynthetic genes within syntenic chromosomal regions of tomato, potato, and pepper. Although the coding region of littorine synthase (LS) has been completely eliminated from these species, promoter remnants remain readily identifiable, while the outgroup species *Iochroma cyaneum* still retains fragments corresponding to the first two exons. Likewise, although CYP80F1 (littorine mutase) has been lost from nearly all non-TA-producing Solanaceous species, a single homolog persists in *Petunia*, and remnants of the first and second exons remain detectable within the syntenic regions of pepper and potato. Together, these genomic relics provide compelling evidence supporting a model of multiple independent and stepwise losses of the tropane alkaloid biosynthetic pathway during Solanaceae evolution [32,39].

### 6.2. Convergent Evolution of Tropane Alkaloid Biosynthesis in Solanaceae and Erythroxylaceae

Solanaceae and Erythroxylaceae diverged more than 129 million years ago and have since evolved independently. Despite their distant evolutionary relationship, both families ultimately construct nearly identical bicyclic tropane scaffolds by integrating polyamine-derived nitrogen-containing precursors with polyketide-derived carbon skeletons. However, from the earliest polyamine reactions through the terminal tailoring steps, virtually every enzymatic transformation is catalyzed by unrelated protein families [40].

For example, Solanaceous species evolved putrescine *N*-methyltransferase (PMT) through neofunctionalization of a duplicated spermidine synthase (SPDS) gene, allowing direct conversion of putrescine into *N*-methylputrescine. In contrast, Erythroxylaceae retained an ancestral metabolic module that had been lost in Solanaceae and instead recruited the bifunctional enzyme EnSPMT1 together with the FAD-dependent amine oxidase EnAOF1 to generate *N*-methylputrescine through sequential spermidine methylation and oxidative aminopropyl removal. Likewise, although both families independently recruited type III polyketide synthases (PKSs) to synthesize the common intermediate 3-oxoglutaric acid, the underlying evolutionary trajectories differ substantially. The Erythroxylaceae-specific enzymes EnPKS1 and EnPKS2 originated through lineage-specific expansion following whole-genome duplication, whereas the Solanaceous PYKS lineage evolved independently from a different ancestral PKS. Thus, parallel recruitment of distinct biosynthetic genes ultimately produced the same metabolic intermediate [40,41].

Perhaps the most compelling evidence for convergent evolution lies in the oxidative cyclization step responsible for assembling the tropane bicyclic scaffold. Although both lineages catalyze the same chemical transformation, they employ entirely unrelated cytochrome P450 enzymes. Erythroxylaceae utilizes the CYP81 enzyme EnCYP81AN15, whereas Solanaceae recruits the CYP82 enzyme CYP82M3. Structural analyses combined with substrate docking have revealed an extraordinary example of topological convergence: EnCYP81AN15 positions the substrate by forming a salt bridge between Asp372 and the pyrrolidine nitrogen from one side of the catalytic pocket, whereas CYP82M3 employs Asp316 together with Arg123 from the opposite side of the active site to achieve essentially the same catalytic outcome. Thus, identical chemical transformations have evolved through fundamentally different active-site architectures [41].

The downstream tailoring reactions likewise illustrate distinct evolutionary solutions. Ecgonone, a key intermediate in cocaine biosynthesis, is highly unstable and readily undergoes spontaneous decarboxylation under physiological conditions. Solanaceous species exploit this intrinsic instability by allowing spontaneous decarboxylation to produce tropinone, which is subsequently reduced by the SDR-family enzymes TRI or TRII to generate tropine or pseudotropine [42]. In contrast, Erythroxylaceae intercepts this unstable intermediate by recruiting the SABATH-family methyltransferase EnMT4, whose active-site residues Glu216 and Ser153 establish a hydrogen-bonding network that rapidly methylates the carboxyl group of ecgonone, thereby preventing spontaneous decarboxylation and preserving the characteristic 2-carbomethoxy substituent of cocaine. The remaining downstream reactions are likewise catalyzed by unrelated enzymes: reduction is performed by the AKR-family enzyme MecgoR [107], whereas the final acylation step is catalyzed by the cytosolic BAHD acyltransferase cocaine synthase (CS) [108]. These enzymes bear no evolutionary relationship to the corresponding Solanaceous enzymes, which instead recruit SDR reductases and the vacuole-associated SCPL acyltransferase littorine synthase (LS). Collectively, these findings demonstrate that Solanaceae and Erythroxylaceae independently assembled entirely different enzymatic toolkits to construct essentially the same complex tropane scaffold, providing one of the clearest molecular examples of convergent evolution in plant specialized metabolism [40].

Key functionally characterized enzymes participating in tropane alkaloid biosynthesis, together with their catalyzed reactions, source organisms and reported subcellular locations, are summarized in Table 1 for quick reference.

## 7. Conclusions and Future Perspectives

Over the past four decades, research on TA has evolved from natural product chemistry and classical biochemistry to molecular genetics, structural biology, and evolutionary genomics. Although substantial progress has been achieved in elucidating TA biosynthetic pathways, the level of experimental validation differs among individual enzymatic steps. Several core reactions, including tropane scaffold formation mediated by PMT, MPO, PYKS, CYP82M3, and stereoselective reduction catalyzed by TRI or TRII, have been supported by biochemical characterization and genetic evidence. In contrast, some proposed regulatory components and pathway-associated enzymes remain incompletely characterized, highlighting the need for further functional validation and mechanistic studies. More importantly, the central objective of the field has undergone a fundamental transition. Early studies primarily sought to elucidate how plants naturally synthesize TA. However, with the progressive completion of biosynthetic pathways, increasing mechanistic understanding of catalytic enzymes, and rapid advances in comparative genomics and evolutionary biology, TA research is entering a new era that extends beyond pathway discovery toward pathway design [30,39,40,41]. Consequently, the ultimate goal of biosynthetic studies is no longer simply to reconstruct complete metabolic maps, but also to uncover the general principles governing biosynthetic evolution, thereby establishing a theoretical framework that enables the rational engineering and redesign of natural product biosynthesis.

One of the major unresolved questions concerns the spatial organization of TA metabolism. Existing evidence indicates that the expression patterns of genes involved in hyoscyamine, scopolamine, and cocaine biosynthesis are frequently uncoupled from the tissues in which these alkaloids ultimately accumulate, suggesting that TA production depends not only on enzyme activities but also on long-distance metabolite transport and intracellular trafficking [110]. Although several transporter families, including ATP-binding cassette (ABC) transporters, multidrug and toxic compound extrusion (MATE) transporters, and proton-coupled transporters, are likely involved, the molecular basis of TA transport remains largely unknown [111,112]. Future studies should therefore prioritize the identification of TA transporters and elucidate their substrate specificity, subcellular localization, and functional interactions with biosynthetic enzymes. Integrating biosynthetic pathways with transport systems and metabolite sequestration mechanisms will likely become a critical strategy for alleviating product toxicity, relieving feedback inhibition, and improving metabolic flux in engineered production platforms. Beyond metabolite transport, another major regulatory layer remains poorly understood. Transcriptional regulation of TA biosynthesis remains poorly understood [113,114]. Comprehensive characterization of transcriptional regulatory networks—including root-specific transcription factors, hormone-responsive regulators (e.g., ABA, jasmonate, and ethylene signaling), and chromatin remodeling factors—will provide important insights into the coordinated regulation of metabolic flux and alkaloid accumulation.

Although most catalytic steps of TA biosynthesis have now been identified, several representative enzymes continue to illustrate important unresolved mechanistic questions. For example, the cytochrome P450 CYP82M3, which has been proposed to catalyze the oxidative cyclization step leading to tropinone formation, has yet to be structurally characterized at high resolution. Determining its substrate recognition mechanism, conformational dynamics, and electron transfer pathway will be essential for understanding tropane ring formation and for guiding rational enzyme engineering [77]. Similarly, CYP80F1 (littorine mutase) exhibits catalytic promiscuity, producing both desired products and side products, highlighting the need for structural biology, molecular dynamics simulations, and structure-guided mutagenesis to improve catalytic specificity and efficiency [99]. Hyoscyamine dehydrogenase (HDH), reported to be partially dispensable owing to compensatory reductive pathways, participates in the conversion of hyoscyamine aldehyde, and genetic studies have suggested the existence of compensatory reductive pathways following HDH silencing. Identification of these alternative reductases will further clarify the robustness and plasticity of the TA metabolic network and may provide additional targets for metabolic engineering and molecular breeding [33].

The increasing demand for medicinal TA, together with the intrinsic limitations of plant cultivation, has stimulated considerable interest in microbial synthetic biology. Significant progress has recently been achieved toward the de novo biosynthesis of hyoscyamine and scopolamine in yeast. Nevertheless, current production levels (approximately 480 μg L^−1^ for hyoscyamine and 170 μg L^−1^ for scopolamine) remain far below those required for industrial application [27,111]. Future engineering efforts should therefore emphasize modular pathway optimization, balanced precursor supply, enzyme engineering, cofactor regeneration, dynamic metabolic regulation, and reduction in cellular metabolic burden. Incorporation of plant-derived transporters and compartmentalization strategies into microbial hosts may further enhance metabolic efficiency and product accumulation.

Traditionally, metabolic engineering of plant natural products has largely followed a “copy-the-natural-pathway” strategy, in which complete biosynthetic pathways are transferred into microbial or heterologous plant hosts [115,116,117]. However, plant metabolic networks are inherently characterized by dispersed metabolic flux, extensive inter-organelle transport, tightly coordinated regulation, and suboptimal catalytic efficiency, making faithful pathway transplantation difficult to achieve. The evolutionary history of TA biosynthesis suggests an alternative paradigm. Independent plant lineages have repeatedly evolved structurally similar tropane alkaloids through distinct enzymatic repertoires and independent metabolic modules. This observation implies that the fundamental objective of synthetic biology should not necessarily be to reproduce natural pathways themselves, but rather to preserve the underlying chemical logic required for target molecule synthesis rather than every individual enzymatic step. Future metabolic engineering may therefore benefit from evolution-guided modular design, in which functionally equivalent enzymatic modules from diverse evolutionary lineages are rationally combined—potentially enabled by AI-driven enzyme mining and genome-wide biosynthetic locus discovery—to construct compact, efficient, and industrially optimized synthetic pathways instead of directly replicating native plant metabolism. The integration of genome mining with artificial intelligence-based approaches provides new opportunities for discovering and optimizing biosynthetic enzymes. Large-scale genomic and transcriptomic resources have facilitated the identification of candidate genes involved in specialized metabolism, whereas protein structure prediction and machine learning approaches may assist in evaluating enzyme function and substrate specificity. However, computational predictions require experimental validation because catalytic performance is strongly influenced by substrate availability, protein flexibility, and cellular context.

More broadly, studies of TA biosynthesis are transforming plant natural product research from pathway-centered research toward the broader concept of metabolic designability. The convergent evolution observed between Solanaceae and Erythroxylaceae, together with repeated lineage-specific pathway losses within Solanaceae, not only explains the highly discontinuous distribution of TAs across angiosperms but also demonstrates the remarkable modularity, plasticity, and evolvability of plant specialized metabolism [40]. Future research should therefore move beyond asking how plants synthesize tropane alkaloids and instead address a more fundamental evolutionary question: how do plants repeatedly evolve novel metabolic solutions? Answering this question will require an integrated theoretical framework linking enzyme neofunctionalization, biosynthetic gene cluster evolution, metabolic network rewiring, and the diversification of specialized metabolites. Such a framework will provide the conceptual foundation for the next generation of rational metabolic engineering.

Ultimately, the future of TA research lies not merely in reconstructing natural biosynthetic pathways but in exploiting the evolutionary design principles shaped over hundreds of millions of years of plant evolution. Biosynthetic pathway elucidation, enzymatic mechanism, evolutionary biology, and synthetic biology should therefore be regarded not as independent disciplines but as complementary pillars supporting the rational engineering of natural product metabolism. Coupled with continuing advances in multi-omics technologies, artificial intelligence, protein design, CRISPR/Cas-based pathway engineering, cell-free biosynthesis, and high-throughput biomanufacturing, these developments are expected not only to overcome the long-standing dependence of medicinal TAs on plant resources but also to extend beyond the constraints of naturally evolved biosynthetic pathways. Ultimately, these advances may enable the production of natural and structurally modified tropane alkaloid derivatives through engineered biosynthetic systems, expanding the chemical diversity accessible by synthetic biology approaches.

## Figures and Tables

**Table 1:** Summary of functionally characterized tropane-alkaloid-biosynthetic enzymes.

Full Enzyme Name	Enzyme Abbreviation	Catalytic Reaction	Source Species Reported	Subcellular Localization
Ornithine Decarboxylase	ODC	Catalyzes the conversion of ornithine to putrescine	*Atropa belladonna*, *Hyoscyamus niger*, *Anisodus luridus*, *Datura innoxia*, *Mus musculus*	No clear report
Arginine Decarboxylase	ADC	Catalyzes the first and rate-limiting step of the ADC-mediated putrescine biosynthetic pathway, converting L-arginine into agmatine through decarboxylation	*Erythroxylum coca*, *Atropa belladonna*, *Arabidopsis thaliana*	No clear report
Agmatine Iminohydrolase	AIH	Catalyzes the second step of the ADC pathway by hydrolyzing agmatine, the product of arginine decarboxylation, into *N*-carbamoylputrescine with the concomitant release of ammonia	*Arabidopsis thaliana*	Mainly localized in the endoplasmic reticulum (ER); AtAIH.4 isoform is localized in chloroplasts
*N*-carbamoylputrescine Amidohydrolase	CPA	Catalyzes the final step of the ADC-mediated putrescine biosynthetic pathway by specifically hydrolyzing *N*-carbamoylputrescine to produce putrescine and ammonia	*Arabidopsis thaliana*	Mainly localized in the endoplasmic reticulum (ER)
Putrescine *N*-methyltransferase	PMT	Catalyzes the transfer of a methyl group from *S*-adenosylmethionine to putrescine, producing *N*-methylputrescine	*Atropa belladonna*, *Nicotiana sylvestris*, *Hyoscyamus muticus*, *Hyoscyamus niger*	Localized in the pericycle
*N*-methylputrescine Oxidase	MPO	Catalyzes the oxidative deamination of *N*-methylputrescine, producing 4-methylaminobutanal, which cyclizes into the *N*-methyl-Δ^1^-pyrrolinium cation	*Atropa belladonna*, *Datura* spp., *Hyoscyamus niger*, *Nicotiana tabacum*	No clear report
Type III Polyketide Synthase	PYKS	Catalyzes the condensation of two malonyl-CoA molecules to form 3-oxoglutaric acid, which undergoes nonenzymatic condensation with the *N*-methyl-Δ^1^-pyrrolinium cation, producing 4-(1-Methyl-2-pyrrolidinyl)-3-oxobutanoic acid	*Atropa belladonna*, *Anisodus acutangulus*	No clear report
Tropinone Synthase	CYP82M3	Catalyzes the oxidative cyclization of 4-(1-methyl-2-pyrrolidinyl)-3-oxobutanoic acid to generate tropinone	*Atropa belladonna*	No clear report
4-Hydroxyphenylpyruvate Aminotransferase 4	ArAT4	Mediates the transamination reaction between phenylalanine and 4-hydroxyphenylpyruvate, leading to the formation of phenylpyruvic acid	*Atropa belladonna*	No clear report
Phenylpyruvic Acid Reductase	PPAR	Facilitates the conversion of phenylpyruvic acid into phenyllactate via a reductive reaction	*Atropa belladonna*	Localized in the pericycle and endodermis cells of lateral roots
Phenyllactate UDP-Glycosyltransferase	UGT1	Catalyzes the glycosylation of phenyllactate to form phenyllactylglucose	*Atropa belladonna*	No clear report
Littorine Synthase	LS	Catalyzes the esterification of phenyllactylglucose with tropine to form littorine	*Atropa belladonna*	No clear report
Littorine Mutase	CYP80F1	Catalyzes the conversion of littorine to hyoscyamine aldehyde via intramolecular rearrangement	*Hyoscyamus niger*	No clear report
Hyoscyamine Dehydrogenase	HDH	Catalyzes the reduction in hyoscyamine aldehyde to hyoscyamine	*Atropa belladonna*	No clear report
Hyoscyamine 6β-Hydroxylase	H6H	Catalyzes the 6β-hydroxylation of hyoscyamine to form anisodamine, which is subsequently epoxidized to scopolamine	*Atropa belladonna*	No clear report
Spermidine Synthase/*N*-methyltransferase	EnSPMT1	A unique bifunctional enzyme that combines spermidine synthase and spermidine *N*-methyltransferase (SMT) activities. It catalyzes the first committed step of the polyamine branch leading to cocaine biosynthesis by generating the methylated precursor required for downstream metabolism	*Erythroxylum coca*	No clear report
FAD-Dependent Amine Oxidase	EnAOF1	Functions as the key oxidative cleavage enzyme within the methylated polyamine pathway. It catalyzes the oxidative degradation of *N*-methylspermidine to generate *N*-methylputrescine	*Erythroxylum coca*	No clear report
*N*-Methylputrescine Oxidase 1	EnMPO1	Catalyzes the oxidative deamination of the terminal primary amino group of *N*-methylputrescine to produce 4-methylaminobutanal. This unstable aldehyde subsequently undergoes spontaneous intramolecular cyclization to generate the *N*-methyl-Δ^1^-pyrrolinium cation	*Erythroxylum coca*	Localized in plant peroxisomes
Type III Polyketide Synthase 1/2	EnPKS1/EnPKS2	Using two molecules of malonyl-CoA as substrates, these enzymes perform only a single condensation cycle to generate 3-oxoglutaric acid, and subsequently undergoes spontaneous, non-enzymatic Mannich condensation with the *N*-methyl-Δ^1^-pyrrolinium cation to produce 4-(1-methyl-2-pyrrolidinyl)-3-oxobutanoic acid	*Erythroxylum coca*	No clear report
EnCYP81AN15	EnCYP81AN15	Catalyzes the oxidative cyclization step responsible for formation of the cocaine tropane skeleton. It selectively catalyzes the oxidative cyclization of the (S)-enantiomer of 4-(1-methyl-2-pyrrolidinyl)-3-oxobutanoic acid to generate the unstable intermediate ecgonone	*Erythroxylum coca*	No clear report
Ecgonone Methyltransferase 4	EnMT4	Catalyzes the methyl esterification step linking tropane ring formation with downstream cocaine biosynthesis. It specifically methylates the carboxyl group of ecgonone to generate methylecgonone	*Erythroxylum coca*	No clear report
Methylecgonone Reductase	MecgoR	Catalyzes the NADPH-dependent stereospecific reduction in methylecgonone to methylecgonine (2-carbomethoxy-3β-tropine), thereby generating the immediate precursor for the final acylation step catalyzed by cocaine synthase	*Erythroxylum coca*	No clear report
Cocaine Synthase	CS	Catalyzes the final step of cocaine biosynthesis by transferring either a benzoyl or cinnamoyl group from the corresponding CoA thioester to the C3 hydroxyl group of methylecgonine (2-carbomethoxy-3β-tropine). This reaction yields cocaine or cinnamoylcocaine, respectively	*Erythroxylum coca*	No clear report

## Data Availability

No new data were created or analyzed in this study. Data sharing is not applicable to this article.

## Abbreviations

The following abbreviations are used in this manuscript:

ABC	ATP-binding cassette
ADC	Arginine decarboxylase
AIH	Agmatine iminohydrolase
AKR	Aldo-keto reductase
ArAT4	*L-phenylalanine*: 4-hydroxyphenylpyruvate aminotransferase 4
At4CL5	*Arabidopsis* 4-coumarate ligase 5
CPA	*N*-carbamoylputrescine amidohydrolase
CS	Cocaine synthase
CuAO	Copper-dependent amine oxidase
CYP80F1	Littorine mutase
CYP82M3	Tropinone synthase
dcSAM	S-adenosylmethionine
DAO	Diamine oxidase
EnAOF1	FAD-dependent amine oxidase
EnMT4	Ecgonone methyltransferase 4
ER	Endoplasmic reticulum
HDH	Hyoscyamine dehydrogenase
H6H	Hyoscyamine 6β-hydroxylase
LS	Littorine synthase
MATE	Multidrug and toxic compound extrusion
MecgoR	Methylecgonone reductase
MPO	*N*-methylputrescine oxidase
MPOA	4-(1-methyl-2-pyrrolidinyl)-3-oxobutanoic acid
OGA	3-oxoglutaric acid
ODC	Ornithine decarboxylase
PAO	Polyamine oxidase
PMT	Putrescine *N*-methyltransferase
PPAR	Phenylpyruvic Acid Reductase
PYKS	Type III polyketide synthase
RNAi	RNA interference
SAM	S-adenosyl-L-methionine
SCPL	Serine carboxypeptidase-like
SDR	Short-chain dehydrogenase/reductase
SMT	Spermidine *N*-methyltransferase
SPDS	Spermidine synthase
TA	Tropane alkaloid
TRI	Tropinone reductase I
TRII	Tropinone reductase II
TS	3β-Tigloyloxytropane synthase
UGT1	Phenyllactate UDP-glycosyltransferase
VIGS	Virus-induced gene silencing
α-WGT	Ancient whole-genome triplication
